# Effectiveness of tailored lifestyle interventions, using web-based and print-mail, for reducing blood pressure among rural women with prehypertension: main results of the *Wellness for Women: DASHing towards Health* clinical trial

**DOI:** 10.1186/s12966-014-0148-2

**Published:** 2014-12-06

**Authors:** Patricia A Hageman, Carol H Pullen, Melody Hertzog, Linda S Boeckner

**Affiliations:** Physical Therapy Education, School of Allied Health Professions, College of Medicine, University of Nebraska Medical Center, Omaha, Nebraska 68198-4420 USA; College of Nursing, University of Nebraska Medical Center, Omaha, NE 68198-5330 USA; College of Nursing, University of Nebraska Medical Center, Lincoln, NE 68588-0220 USA; Nutrition and Health Sciences, University of Nebraska-Lincoln, Lincoln, NE 68583-0806 USA

**Keywords:** Prehypertension, DASH, Rural women, Tailored messages, Lifestyle modification, Web-based, Print-mailed, Community-based, Health promotion model, Physical activity

## Abstract

**Background:**

Lifestyle modification is recommended for management of prehypertension, yet finding effective interventions to reach rural women is a public health challenge. This community-based clinical trial compared the effectiveness of standard advice to two multi-component theory-based tailored interventions, using web-based or print-mailed delivery, in reducing blood pressure among rural women, ages 40–69, with prehypertension.

**Methods:**

289 women with prehypertension enrolled in the *Wellness for Women: DASHing towards Health* trial, a 12-month intervention with 12-month follow-up. Women were randomly assigned to groups using a 1:2:2 ratio, comparing standard advice (30-minute counseling session) to two interventions (two 2-hour counseling sessions, 5 phone goal-setting sessions, strength-training video, and 16 tailored newsletters, web-based or print-mailed). Linear mixed model methods were used to test planned pairwise comparisons of marginal mean change in blood pressure, healthy eating and activity, adjusted for age and baseline level. General estimating equations were used to examine the proportion of women achieving normotensive status and meeting health outcome criteria for eating and activity.

**Results:**

Mean blood pressure reduction ranged from 3.8 (SD = 9.8) mm Hg to 8.1 (SD = 10.4) mm Hg. The 24-month estimated marginal proportions of women achieving normotensive status were 47% for web-based, and 39% for both print-mailed and standard advice groups, with no group differences (p = .11 and p = .09, respectively). Web-based and print-mailed groups improved more than standard advice group for waist circumference (p = .017 and p = .016, respectively); % daily calories from fat (p = .018 and p = .030) and saturated fat (p = .049 and p = .013); daily servings of fruit and vegetables (p = .008 and p < .005); and low fat dairy (p < .001 and p = .002). Greater improvements were observed in web-based versus standard advice groups in systolic blood pressure (p = .048) and estimated VO_2_max (p = .037). Dropout rates were 6% by 6-months, 11.4% by 24 months, with no differences across groups.

**Conclusions:**

Rural women with prehypertension receiving distance-delivery theory-based lifestyle modifications can achieve a reduction of blood pressure and attainment of normotensive status.

**Trial registration:**

ClinicalTrials.gov NCT00580528

## Background

Prehypertension is a distinct classification of blood pressure (systolic blood pressure of 120–139 mm Hg and/or diastolic blood pressure of 80–89 mm Hg) defined by the Joint National Committee on the Prevention, Detection, Evaluation, and Treatment of High Blood Pressure (JNC-7) to identify individuals having a greater risk for developing hypertension (systolic pressure ≥140 mm Hg and/or diastolic pressure ≥90 mm Hg) [1]. Prehypertension is linked with an increased risk of major cardiovascular events independent of other cardiovascular disease risk factors, and nearly one-third of blood pressure related deaths from coronary heart disease occur in individuals with prehypertension [2,3].

Of public health concern, women are frequently unaware of their blood pressure and the challenges that prehypertension or hypertension may pose to their health [4]. A high prevalence of prehypertension is observed among women across the USA and among rural Midwestern women [5-7]. Compared to urban women, rural women have higher rates of abdominal obesity, and obesity-related behaviors such as physical inactivity and poorer diets, which are risk factors for prehypertension [8-10]. Rural women represent one of the largest medically underserved populations in the United States, having limitations in access to health care due to distance and lack of services [11]. Rural women are also less likely to receive preventive measures, such as counseling in diet and activity, compared to their urban counterparts [12,13].

As prehypertension is a modifiable risk factor for cardiovascular disease, the number one cause of death in women, finding methods to implement effective interventions for rural women to reduce blood pressure to optimal levels is warranted.

Lifestyle modification including healthy eating and activity has been recommended as the primary treatment for individuals with prehypertension to achieve optimal blood pressure [1,14]. The PREMIER study found success in reducing blood pressure among individuals with prehypertension or hypertension when using multiple intensive group meetings (up to 2 hours in length) and face-to-face individual counseling sessions to promote lifestyle modifications consistent with the Dietary Approaches to Stop Hypertension (DASH) eating plan and physical activity [15-17]. This intervention format and delivery may not be feasible or practical to reach midlife and older women with prehypertension in rural community settings due to the lack of services and distance to reach services as previously mentioned.

Distance-delivery methods for implementing lifestyle modification interventions show promise for reaching rural women. A community-based randomized clinical trial, called the *Wellness for Women* trial, conducted by the same team of investigators prior to this *Wellness for Women: DASHing towards Health* trial, found that midlife and older rural Midwestern women were receptive to receiving lifestyle behavior-change interventions for healthy eating and activity via print-mailed newsletters and that women were willing to participate in the 24 month trial with low overall attrition (4.5%) [18,19]. That trial tested tailoring of the intervention, whereby computer-generated messages were developed to present in clear detail only the most relevant information for each woman, based on data that she provided in responses to questionnaires at the assessments. The advantage of tailoring is that it offers an individualized approach, similar to that provided in individual face-to-face interactions, with studies showing tailored materials are more likely to be read and remembered as well as to motivate individuals for change [20,21]. In the *Wellness for Women* trial, a higher proportion of women who received theory-based tailored newsletters met healthy eating and activity targets after the 12-month intervention and at 12-month post-intervention follow-up than did those who received generic (non-tailored) newsletters [18,19].

Lifestyle modification interventions that incorporate web-based components offer advantages of providing tailored messages at low cost in order to reach a large audience across great distances, with convenience for the users. Emerging evidence suggests that web-based interventions may be effective for offering lifestyle modification interventions for eating and activity. Web-based interventions are often augmented with a multi-component approach that may include self-monitoring and/or individual or group counseling via face-to-face, telephone, or Internet [22-24].

Few community-based studies were found that used web-based tailored lifestyle modification interventions for healthy eating and activity in individuals with prehypertension. Compared with this clinical trial, other studies used different approaches and populations and were of shorter duration (10 weeks to 6 months) [25-27]. Bavikati et al. [25] used a computerized participant management system to implement a 6-month comprehensive therapeutic lifestyle change intervention using computer generation of risk factor goals and action plans and behaviorally oriented counseling by face-to-face, or via telephone and the Internet. Bavikati and associates targeted behavior change for healthy eating and activity plus smoking cessation and weight- and stress-management in an ethnically diverse population (n = 2,478) of men and women with prehypertension, with a finding that 38.4% (n = 952) of participants normalized their blood pressure during the study [25]. Dorough et al. [26] conducted a 10-week intervention study of 27 adults with prehypertension, ages 45–65, who received a 60-minute counseling session with a dietitian and then were randomized to either standard advice or web-based intervention groups. Findings indicated that individuals in the web-based intervention group who received tailored communications had more desirable results. Watson et al. [27] evaluated a 6-month web-based self-management intervention among 404 employees (mean age =50, SD =8) that included self-monitoring of blood pressure for the control group and self-monitoring of blood pressure plus automated tailored messages in the intervention group. Post-intervention, they reported higher proportions of individuals with prehypertension attaining a decline of ≥10 mm Hg in SBP and ≥5 mm Hg in DBP than those in the control group.

Though Internet penetration has grown in rural areas [28] and implementation of community-based web-delivered interventions has been shown to be feasible in midlife and older rural Midwestern women [29], no studies were found that used web-based delivery for tailored lifestyle modification for rural women with prehypertension or that examined the sustainability of the intervention outcomes. Gaps in the literature remain related to the effectiveness of lifestyle modification for achieving short-term and long-term blood pressure reduction in rural women with prehypertension.

The importance of this research is that it addresses prehypertension, a significant public health challenge, in an understudied population of rural Midwestern women who have documented health disparities (e.g. high prevalence of obesity, a lack of preventative services or counseling, and unique environmental barriers to healthy eating and activity) [8-10]. In recognition of these disparities, the National Institutes of Health convened a workshop to develop a research agenda and recommendations for cardiovascular disease prevention research and practice in high-risk rural communities, recommending that rural women are included as designated priority population by agencies within the US Department of Health and Human Services [30]. This study is unique as it incorporates a theory-based behavior change intervention, using evidence-based strategies (e.g. self-monitoring, tailored messages) and distance-delivery methods within the constraints of a rural environment that has limited, but growing broadband capacity. As maintaining behavior change over time is recognized as a greater challenge than adopting behavior change, this study offers a longer intervention and follow up as recommended by the Institute of Medicine for realistic long-duration outcomes [31].

The purpose of the *Wellness for Women: DASHing towards Health* clinical trial was to compare the effectiveness of standard advice to two multi-component theory-based tailored interventions, using web-based or print-mailed delivery, in reducing blood pressure among midlife and older rural women with prehypertension. The standard advice group received one 30-minute session with a local extension educator, and the intervention groups received two 2-hour sessions with a local extension educator, five phone goal-setting sessions, a strength-training video with elastic bands, and 18 tailored newsletters, delivered as either web-based or print-mailed. If theory-based distance-delivery interventions are effective, they may provide cost-effective alternatives for managing prehypertension in accordance with trends for addressing population health.

Pender’s Health Promotion Model (HPM), founded on the theoretical underpinnings of social cognitive theory, provided the conceptual framework for explaining and modifying the adoption and maintenance of healthy eating and activity behaviors in this study [32]. Four behavior-specific cognitions from the HPM (perceived benefit to action, perceived barriers to action, self-efficacy and interpersonal influences for action), as well as a commitment to a plan of action through goal setting, were used in the development of the tailored interventions.

Specifically, this community-based randomized clinical trial consisted of a 12-month intervention period with a 12-month follow-up to evaluate the effectiveness of: 1) standard advice, 2) an HPM-tailored web-based multi-component intervention, and 3) an HPM-tailored print-mailed multi-component intervention for achieving and maintaining improvements in blood pressure and healthy eating and activity behavioral marker and biomarkers. For measures of blood pressure and healthy eating and activity behavioral markers and biomarkers, the outcomes were reported as 1) change from baseline to each of the subsequent assessments (6 months, 12 months, 18 months, and 24 months) and 2) at each of the four subsequent assessments, the proportion of women who had achieved normotensive status (<120 mm Hg for systolic blood pressure and <80 mm Hg for diastolic) and met health outcome criteria for eating and activity based upon established guidelines from the DASH and American College of Sports Medicine (ACSM) position stand on exercise and hypertension [33,34]. We hypothesized that each of the two intervention groups would have better outcomes than the group receiving standard advice. There was speculation that the web-based tailored intervention might have an incremental effect on women’s improvement in outcomes compared to those in the print-mailed intervention, yet the evidence at the time of trial development was insufficient to state that as a directional hypothesis.

## Methods

The Institutional Review Board of the University of Nebraska Medical Center approved this study (approval # 352-05-FB). Using the standardized and accepted protocol, written informed consent was obtained from all participants prior to their enrollment. The study was conducted between April 2007 and May 2010, with recruitment, enrollment and baseline assessments occurring over one year (April 2007 to April 2008).

### Study participants

The target population were generally healthy women, ages 40–69, from central Nebraska, who met rural status as defined by the Rural Urban Commuting Area (RUCA) codes, having less than 50,000 residents (US Department of Agriculture (USDA) [35]. The four category RUCA classified rural based upon population density and population work commuting patterns, defining large rural with 10,000-49,000 residents, small rural with 2,500-9.999 residents, and isolated rural (<2,400 residents). Women were eligible for the study if they were not taking antihypertensive medication, had a documented blood pressure in the prehypertensive range, and were willing to drive to a centrally-located Health District office for all required visits, either assessment or training.

Other inclusion criteria included being able to speak and read English, communicate over the phone, have ability to use a computer with minimal assistance, walk without an assistive device or oxygen, and have access to the Internet and either a VCR or DVD player. Eligible women were included if they answered “no” to all questions on the Physical Activity Readiness Questionnaire (PAR-Q) or obtained medical clearance from their physician to participate [36]. Major exclusion criteria were: taking medication affecting blood pressure, including antihypertensive, diuretics, or cortisone; self-reported consumption of more than 14 alcoholic drinks per week; enrollment in a formal program of cardiac rehabilitation, undergoing physical rehabilitation, or participating in current cancer treatment; unable to walk one mile continuously without stopping to rest; and/or presenting with other physical or medical restrictions that would preclude following the JNC 7 recommendations [1] for moderate physical activity and healthy eating. Smokers were permitted to enroll with smoking status noted.

### Trial conduct

Participants were recruited through advertisements and press releases in local newspapers for women to participate in free community-based blood pressure screenings in various locations including the YMCA, shopping malls, craft fairs, and county fairs. A special effort was made to recruit and retain Hispanic women, the predominant underrepresented minority group in the region (4% at the time), through contacts at local community action agency sites and churches. At screening sites, women with blood pressure readings that were from 10 mm Hg below to 10 mm Hg above the prehypertensive systolic range or 5 mm Hg below to 5 mm Hg above the prehypertensive diastolic range were told briefly about the study. If interested, they were asked to provide their phone number and address for mailing study information and for a follow-up screening interview.

**Figure 1:**
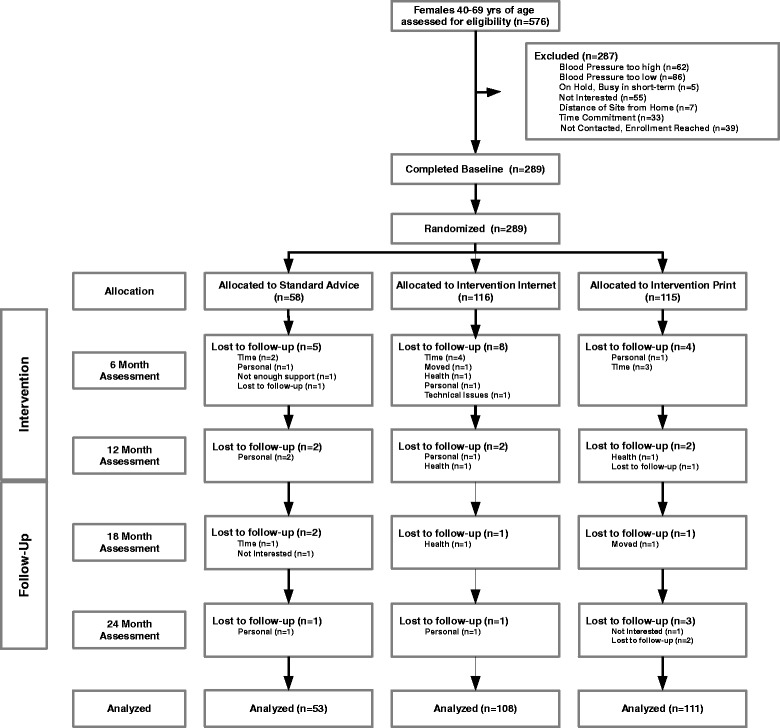
**Participant Flow Diagram of the** ***Wellness for Women: DASHing towards Health*** **Clinical Trial.**

The purpose of the follow-up screening interview was to establish eligibility for the study and provide information so that the women could provide informed consent to participate. The name, address, and phone number of each eligible woman who consented was forwarded to the research nurses at the project office. The research nurses contacted the women to verify if physician clearance was obtained (if needed), and to schedule appointments to complete the consent process and to confirm prehypertensive status with two readings at two visits, an average of four measures, over a one-week period. Once prehypertensive status was confirmed, the baseline assessment was begun. Anytime in the screening processes and/or after enrollment, if a woman had a blood pressure that was hypertensive, the woman was referred to her health care provider for follow-up. These women, if prescribed antihypertensive medication, were permitted to continue participation in their assigned groups and their data were included in the primary analysis, which followed intent-to-treat guidelines in order to preserve the unbiased nature of the groups’ composition. Because the probability of using antihypertension medications could have been related to the intervention received and because such use would have affected certain study outcomes, a supplemental analysis omitting these women was conducted, as described in the analysis section. Participant flow through the trial is shown in Figure 1.

### Randomization

Once consented, each woman was given a sealed envelope which contained her group assignment. Assignments were made according to an allocation schedule previously created by the project statistician, who randomly assigned participants to intervention groups in a 2:2:1 ratio within blocks of 25. The statistician did not have any contact with the women during the trial. The research nurses, who conducted the assessments for the women during the study, were blinded to group assignment. Women were asked not to share their group assignment with the nurses.

### Interventions

#### Standard advice only

Those assigned to the standard advice group received one 30-minute introductory education session at the local extension office from an extension educator who was a registered dietitian. Instruction included a discussion of lifestyle factors that affect blood pressure. The educator outlined the dietary and the physical activity recommendations of DASH and the ACSM position stand on exercise and hypertension, including a discussion about behavioral targets for activity [33,34]. The educator reviewed key behavioral targets and helpful tips to achieve these targets for activity (≥150 minutes weekly), percent calories from saturated fat (<27% daily), fruit and vegetable servings (>8 servings daily), low-fat dairy servings (>2 servings daily), and daily sodium (<2400 mg daily). Women received printed educational materials and had no further contact with the extension educator.

#### Intervention groups

Following baseline assessment, women assigned to the intervention groups (web-based or print-mailed) completed two 2-hour training sessions with instruction by the extension educator/registered dietitian. The content of the first session included introductory education about hypertension and prehypertension, the recommendations from DASH, and the ACSM position stand on exercise and prehypertension, with discussion of strategies to achieve the healthy eating and activity behavioral targets [33,34]. Each woman received a home blood pressure monitor (Omron HEM-780, Omron Manufacturers of America, 3632 Stern Ave, Saint Charles, IL 60174–5406) and a pedometer (Lifestyles SW 200, New Lifestyles Inc. 5201 NE Maybrook RD, Lees Summit MO 64064–1122) with instructions for their use. Women were asked to practice taking their blood pressure daily at the same time of day and to use their pedometer daily during the two-week period prior to attending the second session. The women received instructions to bring their devices as well as their paper logs of eating, activity, and blood pressure to the second session so the educator could review the procedures and answer any questions.

Women assigned to web-based or print-mailed interventions were separated by group for their second educational section. For both groups, the second session reviewed procedures for self-monitoring and included instructions for the women to monitor eating and activity daily and to monitor their blood pressure weekly during the study. The session focused on how to set individual goals for self-monitoring and healthy eating and activity that were specific, measurable, achievable, realistic/relevant, and time-bound. Women assigned to the web-based group received training and practice for accessing the *Wellness for Women: DASHing towards Health* website using their personal IDs and passwords. Instruction included an orientation to the website and instruction in creating log entries for self-monitored blood pressure, eating, and activity. Women received contact information for technical assistance. Women in the print-mailed intervention group received instruction in tracking of their blood pressure, eating, and activity using paper logs.

Commitment to a plan of action occurred through individual telephone goal-setting counseling with women from a trained project counselor at baseline, 3 months, 6 months, 9 months, and 12 months. This counselor was an experienced extension educator/dietitian from the local area, who received two hours of on-site formal training from a senior extension faculty member from the regional office. The project counselor received a training manual and she was instructed to follow a structured telephone script modeled after the Five A’s clinical counseling approach supported by the US Preventive Services Task Force [37]. Counselor adherence to the goal-setting counseling script was monitored using a voice-activated (VOX) telephone cassette recorder that records when it receives sound. Both the counselor and women were notified that the call was being monitored and recorded for quality assurance purposes. The first five tapes and 5% chosen at random each month thereafter underwent reviews conducted by the senior extension faculty member, with any noted problems addressed with the counselor. Once reviewed, the tapes were kept in a locked file cabinet housed in a secured office at the University.

With the exception of web-based or print-mailed delivery, the two theory-based intervention groups were similar in receiving HPM-tailored newsletter content. The 18 newsletters, delivered every two weeks (via web or print-mailed) for the first 6 months (n = 12) and monthly over the second 6 months (n = 6), included both content-specific information and tailored behavioral messaging. The content-specific information included instruction about healthy eating and activity for blood pressure reduction. The tailored messages, web-based or print-mailed, were based on a woman’s personal responses to behavioral surveys administered at baseline, 3 months, 6 months, and 9 months. The web-based delivery offered enhancements such as visual display of feedback from log entries, self-assessment quizzes, and video-demonstrations of physical activities.

After the 3 month assessment, women in the two intervention groups received elastic resistance bands and an instructional exercise video in their preferred format, videotape or DVD. This resistive exercise video used demonstrations by midlife women for the purpose of role-modeling to enhance self-efficacy. Women received messages of encouragement to complete this home resistance training program at least twice per week and to progress to higher levels of band resistance when they could easily perform 15 repetitions of an exercise. The provision of resistance training resources was delayed until the 3 month assessment due to the high participant burden for initiating behavior change for the prescribed healthy eating and activity interventions at baseline.

### Data collection

#### Assessments and measurements

Blood pressure was collected by trained research nurses at seven time points (baseline, 3 months, 6 months, 9 months, 12 months, 18 months and 24 months). Women were required to drive to the project office for two visits, one week apart, at each time point to adhere to the blood pressure protocol. The outcome measures for healthy eating and activity behavioral markers and biomarkers were assessed at baseline, 6 months, 12 months, 18 months (except for lipids), and 24 months. Surveys related to the HPM behavioral determinants were administered to all groups at baseline and 3, 6, and 9 months for the purpose of developing tailored messages for women assigned to the two intervention groups.

#### Blood pressure and resting heart rate

Resting heart rate, using the radial pulse for 60 seconds, and blood pressure were measured following five minutes of quiet sitting. Standardized ausculatory methods were followed, using a calibrated mercury sphygmomanometer with an appropriate size cuff for each woman [38]. At least two blood pressure measurements were obtained at each visit, separated by at least 30 seconds. Systolic blood pressure was the appearance of the first Korotokoff sound and diastolic blood pressure was the disappearance of Korotkoff sounds. For each visit, the blood pressure recorded was the mean of the two measurements that were within five mm Hg for both systolic and diastolic measures. For each assessment point, the recorded blood pressure was the mean of the two final blood pressure measurements from the two visits. Women were asked to avoid caffeine, exercise and smoking for at least 30 minutes prior to their scheduled blood pressure measurements.

#### Eating

The Web version of the 1998 Block Health Habit and History Questionnaire (HHHQ) was used to measure the usual self-selected diets of the women [39]. Paper/pencil versions of the HHHQ have been previously validated [40,41]. Pearson product moment correlations between the Web and paper/pencil versions of the HHHQ produced correlation coefficients ranging between .54 and .86 for the dietary variables (median r = .80), were statistically significant at < .05, and provided evidence of parallel forms reliability of the web-based version [39]. This survey asked for the frequency of consumption of particular food items during the last three months. The survey provided estimates of daily caloric intake, % of dietary calories from fat, saturated fat, daily servings of food groups, and daily sodium intake.

#### Physical activity

The Modified 7-Day Activity Interview instrument was used to measure time engaged in moderate or greater intensity activity, capturing the woman’s self-reported time spent and her perceived intensity of activity for a variety of activities, including strengthening exercise, and these data were used to calculate daily estimated energy expenditure [42]. The instrument yields data concerning hours per day spent in sleep and in light, moderate, hard and very hard activity (based on MET levels), total kilocalories per kilogram of body weight expended per day and total calories expended per day. Hellman et al. [42] assessed construct validity of the Modified 7-Day Activity Interview with findings that its scores discriminated (z = −6.14) between active and inactive groups, and were correlated (rho =0.62) with perceived levels of physical activity among community-dwelling older adults. In a separate study, the Modified 7-Day Activity Interview scores correlated with estimated VO_2_max (rho = .47) among cardiac rehabilitation participants [43]. The instrument was found to be sensitive to change with rural women ages 50–69 who participated in a 12 month healthy eating and activity tailored newsletter intervention [29].

Cardiorespiratory fitness was estimated using the submaximal one-mile walk test whereby each woman was asked to walk as fast as possible over a one-mile indoor track. Estimated VO_2_max was calculated using Kline’s equation developed for women using variables of weight, age, total walk time, and 15-second post-activity heart rate [44]. A cross-validation analysis that tested a large heterogeneous sample of 178 women and 165 men reported the one-mile walk test produced a valid submaximal assessment of estimated VO_2_max [44]. The predictive validity of this equation for women over 65 years also has been substantiated [45]. Among women ages 50–69, high test-retest reliability was observed for estimated VO_2_max using the gender specific and generalized equations (ICCs [3,1] =0.97 and 0.96 respectively) and for one-mile walk times (ICC = 0.96) across a two week period [46]. In a separate study, estimated VO_2_max as calculated from the one-mile walk test was sensitive to change in women ages 50–69 following a three month web-based physical activity intervention [29].

#### Biomarkers affected by activity and eating

Body composition measures used for the main results of this trial were measured using body mass index and waist circumference. This field trial used the Tanita scale [TBF-215, Tanita Corporation of America, Inc., 2625 S. Clearbrook Dr., Arlington Heights, IL 60005–9824] for measurement of height and weight, with body mass index (BMI) being calculated as weight in kilograms divided by height in meters-squared [47]. A tape measure was used to assess waist circumference, where the tape was placed snug and parallel to the floor without skin compression in a horizontal plane around the abdomen at the level of the iliac crest. The measurement was taken at the end of expiration, with the average of two trials recorded [48].

Blood specimens were drawn from women who completed a 12-hour fast, using standardized venipuncture methods by research nurses trained by an American Society for Clinical Pathology clinical laboratory scientist. The nurses centrifuged the blood after appropriate time for clotting, 30 to 60 minutes, with blood serum separated, frozen, and shipped to the rapid response laboratory at the university where it was processed to determine total cholesterol, low-density lipoprotein (LCL-C), high-density lipoprotein (HDL-C), triglycerides, and fasting glucose following a standardized protocol [49].

#### Measurement of behavioral determinants for tailoring

Women completed validated web-based surveys to assess their perceptions about the Health Promotion Model behavioral determinants related to benefits and barriers to, and interpersonal support and self-efficacy for, healthy eating and activity. Selected items from these survey measures were then used as a basis for tailoring messages for the two intervention groups following a methodology similar to our prior *Wellness for Women* clinical trial [18]. The instruments included the Exercise Benefits/Barriers Scales, Healthy Eating Benefits/Barriers Scales, Self-efficacy for Eating and Exercise Habits Scales, Family and Friend Support for Exercise Habits Scales, and Family and Friend Support for Healthy Eating Habits Scales [18,50,51]. The survey results will be reported in a future paper.

#### Sample size

Sample size was based on power analyses for planned comparisons of each intervention group with the standard advice group (one-sided tests) and of the two intervention groups (two-sided test) on the endpoints of average change in blood pressure, eating and activity behavioral markers, and biomarkers. Changes in a range of blood pressure, diet, and physical activity measures among prehypertensive women in a preliminary study were used as the basis for effect size estimates: a difference of .4 standard deviations between intervention and advice-only groups, .5 standard deviations between the two intervention groups. With 92 subjects in each intervention group and 42 subjects in the standard advice group, estimated power was ≥ .80 for each test using alpha = .05. The target sample size for recruitment was 110 participants per intervention group to allow for 20% attrition and 55 women for the standard advice group to allow for 30% attrition.

#### Data analysis

Descriptive and linear mixed model analyses were performed using IBM SPSS Statistics version 19; SAS 8.3 was used for analyses involving generalized estimating equations. For blood pressure, as well as eating and activity behavioral markers and biomarkers, the primary endpoints were change from baseline, calculated by subtracting the baseline value from later values, so that a negative score indicates a decrease. Groups were compared on average change in outcomes using linear mixed model methods that allow for modeling of within-subject correlation and for inclusion of partial cases. Because the endpoints were change from baseline and data were not imputed, cases having only baseline data were not included. In other respects, the analysis was consistent with the intention-to-treat principle and all participants were analyzed in the groups to which they were assigned. Analyses included an adjustment for baseline levels of the outcome and for participant age. The covariance pattern model that was fit treated time as categorical and estimated an unstructured covariance matrix of within-subject errors.

Dichotomous variables were created for each follow-up time point, indicating the proportion of women who met the health outcome of achieving normotensive status and the proportion of women who met each of the eating and activity criteria (e.g., <27% daily caloric intake from fat). Generalized estimating equations were used to compare the average proportion of women meeting criteria across the 24-month trial, accounting for the non-independence of the observations across time. Time was treated as categorical, an unstructured covariance matrix was specified for within-subject errors, and age and baseline level of outcome were included as covariates. Consistent with the a priori hypotheses, all pairwise tests of web-based or print-mailed compared to standard advice were one-tailed and tests comparing web-based and print-mailed were two-tailed. Odds ratios with 95% confidence intervals were also computed for each pairwise comparison at each follow-up time point using the observed data. All tests used α = .05.

A supplemental analysis, omitting women who were prescribed antihypertension medication during the course of the study, was conducted for the outcomes most directly affected by such medications: systolic and diastolic blood pressure and achievement of normotensive status. Because of the close similarity of results of the two analyses, only the intent-to-treat analysis will be reported in detail below, along with a brief summary highlighting any differences found in the supplemental analysis.

## Results

Two-hundred eighty nine women with mean (SD) ages of 56.4 (6.3) enrolled in the study. Based upon BMI, 53 (18.4%) were normal weight, 109 (37.7%) were overweight and 127 (43.9%) were obese. Baseline characteristics showed the majority of women were Caucasian (97.9%; n = 283) and were employed full-time (60.6%; n = 175); 118 (40.8%) reported having a college degree or higher education. The majority of women reported having a household income > $40,000, with 79 (27.3%) reporting a household income of $40,000-59,000, and 127 (43.9%) reporting a household income of > $60,000. There were 51 (17.6%) living in rural isolated areas, with 208 (71.2%) living in an area defined as large rural. Baseline characteristics by randomized group are found in Table 1.

**Table 1:** **Characteristics of Rural Women with Prehypertension by Randomized Group**

	**Standard Advice**		**Web**		**Print**	
	**n = 58**		**n = 116**		**n = 115**	
**Variable**	***n***	**%**	***n***	**%**	***n***	**%**
**Race or Ethnicity**						
White	57	98%	115	99%	111	97%
Hispanic	0	0%	1	1%	2	2%
Other	1	2%	0	0%	1	1%
No Response	0	0%	0	0%	1	1%
**Education**						
High School or lower	11	19%	21	18%	20	17%
Some College	20	34%	47	41%	52	45%
College Grad or Above	27	47%	48	41%	43	37%
**Employment**						
Full Time	33	57%	73	63%	69	60%
Part Time	12	21%	19	16%	22	19%
**Household Income**						
**<**$20,000	4	7%	10	9%	6	5%
$20,000 to $39,999	14	24%	25	22%	23	20%
$40,000 to $59,999	18	31%	30	26%	31	27%
$60,000 or higher	22	38%	51	44%	54	47%
**Rural Residency**						
Large Rural	39	67%	84	72%	85	74%
Small Rural	7	12%	14	12%	9	8%
Isolated	12	21%	18	16%	21	18%
**Comorbid Conditions**						
Diabetes	5	9%	4	3%	7	6%
Coronary Artery Disease	0	0%	0	0%	0	0%
Heart Attack	0	0%	2	2%	1	1%
Stroke	0	0%	1	1%	2	2%
Emphysema	0	0%	1	1%	2	2%
Asthma	5	9%	13	11%	8	7%
Arthritis	16	28%	26	22%	25	22%
Osteoporosis	7	12%	8	7%	8	7%
Broken hip	0	0%	0	0%	1	1%
Cancer	2	3%	2	2%	4	3%
**Smoke Cigarettes**	0	0%	8	7%	8	7%
**General Health Categorized**						
Excellent to Very Good	35	60%	73	63%	65	57%
Good	20	34%	38	33%	43	37%
Poor to Fair	3	5%	5	4%	7	6%
**BMI Category (kg/m** ^**2**^ **)**						
Normal (< 25)	8	14%	30	26%	15	13%
Overweight (25-29.9)	22	38%	40	34%	47	41%
Obese (≥ 30)	28	48%	46	40%	53	46%

Of the 289 women initially enrolled in the study, 17 (6%) dropped out after completing baseline but before the 6-month data collection, with dropout rates not significantly different across the three groups [χ^2^ (2, N =289) =2.20, p = .333]. Compared to those in the analysis sample, these women had higher BMI, waist circumference, daily caloric intake, and sodium intake, with lower fitness, activity levels, and fruit and vegetable servings, although differences were significant only for BMI [t(287) = −2.01, p = .045] and fruit and vegetable servings [t(287) =2.03, p = .043]. Sample sizes at 24 months were 48, 104, and 104 women, in the standard advice, web-based, and print-mailed groups, respectively. A total of 33 women dropped between baseline and 24 months for an overall attrition rate of 11.4%. Of those who dropped from the study, 30% (n = 10) cited the time commitment as their reason for withdrawal. See Figure 1 for flow diagram.

Within-group distributions of all change variables were examined for outliers and non-normality. All distributions were symmetric, but change in BMI, calories expended, and minutes in moderate or more intense activity tended to have high positive kurtosis. Using a cutoff of *z* > |4.0|, 5–6 cases were identified as outliers for calories expended and minutes of at least moderate intensity activity, with 1 or 2 extreme cases identified for BMI, percentage of fat, percentage of saturated fat, sodium, dairy, and estimated VO_2_max. Analyses of change in outcomes by group were repeated without these outliers. Normality of the distributions improved, but for all but one comparison (discussed later in the text), conclusions did not change when outliers were removed, and these additional analyses will not be presented.

### Change in behavior and biomarkers

Descriptive statistics (group means and standard deviations) for blood pressure and healthy eating and activity behavioral markers and biomarkers at baseline and for change from baseline to 6, 12, 18, and 24 months are presented in Tables 2 and 3. From baseline to the end of the intervention period of 12 months, mean (SD) reductions in systolic blood pressure were 5.6 (10.6) mm Hg in the standard advice group, 7.4 (9.5) mm Hg in the web-based group, and 7.2 (8.5) mm Hg in the print-mail group. Corresponding diastolic reductions were 3.6 (6.6), 4.6 (6.3), and 3.8 (5.7) mm Hg in the standard advice, web-based, and print-mailed groups, respectively. At 24 months (12 months post-intervention), mean (SD) reductions in the standard advice, web-based, and print-mailed groups were 4.1 (9.5), 5.2 (11.7), and 3.8 (9.8) mm Hg, respectively, for systolic blood pressure, and 5.0 (6.1), 5.0 (6.7), and 3.4 (6.8) mm Hg, respectively, for diastolic blood pressure.

**Table 2:** **Mean (SD) change from Baseline** ^**a**^ **for Blood Pressure and Eating and Activity Biomarkers by Group**

	**Observed Means**			**Estimated Mean Difference over 24 months** ^**c**^		
**Variable**	**Standard Advice (SA)**	**Web-based (W)**	**Print-mailed (P)**			
	**n=53** ^**b**^	**n=108** ^**b**^	**n=111** ^**b**^	**Pairwise**	**p-value** ^**d**^	**Estimate (95% CI)**
	**Mean (SD)**	**Mean (SD)**	**Mean (SD)**	**Comparison**		
Systolic blood pressure (mm Hg)						
Baseline	128.3 (6.5)	127.1 (6.7)	128.3 (5.6)	W vs. SA	0.048	-2.1 (-4.6 to 0.4)
Change at 6 months (SD)	-5.1 (10.5)	-6.5 (7.8)	-6.1 (8.2)	P vs. SA	NS	-0.9 (-3.4 to 1.6)
Change at 12 months (SD)	-5.6 (10.6)	-7.4 (9.5)	-7.2 (8.5)	W vs. P	NS	-1.2 (-3.2 to 0.8)
Change at 18 months (SD)	-4.8 (12.2)	-8.1 (10.4)	-6.6 (8.2)			
Change at 24 months (SD)	-4.1 (9.5)	-5.2 (11.7)	-3.8 (9.8)			
Diastolic blood pressure (mm Hg)						
Baseline	76.6 (6.8)	77.3 (6.7)	76.9 (6.6)	W vs. SA	NS	-0.2 (-1.7 to 1.3)
Change at 6 months (SD)	-3.7 (7.5)	-3.8 (5.9)	-3.4 (5.9)	P vs. SA	NS	0.3 (-1.2 to 1.8)
Change at 12 months (SD)	-3.6 (6.6)	-4.6 (6.3)	-3.8 (5.7)	W vs. P	NS	-0.5 (-1.7 to 0.8)
Change at 18 months (SD)	-5.7 (7.7)	-6.1 (7.2)	-5.2 (6.6)			
Change at 24 months (SD)	-5.0 (6.1)	-5.0 (6.7)	-3.4 (6.8)			
BMI (kg/m^2^)						
Baseline	29.7 (4.7)	28.6 (5.1)	30.5 (5.4)	W vs. SA	0.047	-0.4 (-1.0 to 0.1)
Change at 6 months (SD)	-0.6 (1.2)	-0.8 (1.0)	-0.9 (1.4)	P vs. SA	NS	-0.1 (-0.6 to 0.4)
Change at 12 months (SD)	-0.3 (1.6)	-0.8 (1.3)	-0.6 (1.9)	W vs. P	NS	-0.3 (-0.7 to 0.1)
Change at 18 months (SD)	-0.2 (1.4)	-0.7 (1.4)	-0.4 (2.5)			
Change at 24 months (SD)	-0.2 (1.4)	-0.8 (1.7)	-0.2 (3.0)			
Waist circumference (cm)						
Baseline	97.3 (11.2)	95.2 (11.9)	99.4 (13.0)	W vs. SA	0.017	-1.6 (-3.1 to -0.1)
Change at 6 months (SD)	-1.2 (3.8)	-1.9 (3.9)	-2.6 (5.0)	P vs. SA	0.016	-1.6 (-3.1 to -0.1)
Change at 12 months (SD)	-1.5 (5.0)	-3.2 (4.4)	-3.6 (5.4)	W vs. P	NS	-0.02 (-1.2 to 1.2)
Change at 18 months (SD)	-0.5 (4.2)	-2.1 (4.8)	-2.6 (6.8)			
Change at 24 months (SD)	-1.0 (4.1)	-2.4 (4.8)	-1.8 (7.6)			
Estimated VO_2_ max (mg/ml/min)						
Baseline	20.8 (7.7)	22.6 (7.0)	21.8 (7.4)	W vs. SA	0.037	0.9 (-0.1 to 1.8)
Change at 6 months (SD)	1.9 (3.5)	2.3 (2.4)	2.4 (3.1)	P vs. SA	NS	0.4 (-0.6 to 1.3)
Change at 12 months (SD)	2.0 (4.2)	1.9 (3.2)	2.0 (3.5)	W vs. P	NS	0.5 (-0.3 to 1.3)
Change at 18 months (SD)	1.7 (3.9)	2.5 (3.0)	2.1 (3.8)			
Change at 24 months (SD)	0.9 (4.6)	1.7 (3.0)	1.1 (3.6)			
Total cholesterol (mg/dL)						
Baseline	194.7 (32.9)	201.9 (34.8)	194.9 (33.9)	W vs. SA	0.215^e^	2.53 (-3.75 to 8.81)
Change at 6 months (SD)	1.0 (21.5)	1.7 (27.1)	-0.5 (24.6)	P vs. SA	0.375	-1.02 (-7.29 to 5.25)
Change at 12 months (SD)	1.6 (19.1)	-0.04 (23.7)	-1.8 (22.3)	W vs. P	0.168	3.55 (-1.51 to 8.60)
Change at 24 months (SD)	3.3 (27.3)	6.8 (30.0)	4.5 (30.9)			
HDL (mg/dL)						
Baseline	57.3 (12.7)	58.3 (15.4)	56.0 (12.5)	W vs. SA	0.155	1.01 (-.95 to 2.98)
Change at 6 months (SD)	-3.6 (7.8)	-2.6 (7.6)	-2.8 (6.6)	P vs. SA	0.163	0.98 (-.98 to 2.95)
Change at 12 months (SD)	-2.4 (7.4)	-1.5 (8.6)	-1.1 (7.1)	W vs. P	0.968	.03 (-1.55 to 1.62)
Change at 24 months (SD)	-5.7 (7.7)	-5.5 (9.1)	-5.0 (7.0)			
LDL (mg/dL)						
Baseline	114.7 (27.0)	118.5 (32.2)	115.0 (29.6)	W vs. SA	0.264^e^	1.79 (-3.78 to 7.37)
Change at 6 months (SD)	1.4 (17.5)	4.0 (24.6)	3.1 (21.7)	P vs. SA	0.455	-0.32 (-5.89 to 5.25)
Change at 12 months (SD)	2.3 (16.8)	1.2 (20.2)	0.6 (20.3)	W vs. P	0.356	2.11 (-2.39 to 6.61)
Change at 24 months (SD)	11.5 (19.0)	13.6 (25.8)	10.8 (27.1)			
Triglycerides (mg/dL)						
Baseline	113.5 (55.1)	127.5 (72.3)	119.4 (57.4)	W vs. SA	0.114	-6.2 (-16.4 to 3.9)
Change at 6 months (SD)	16.1 (43.5)	1.3 (43.4)	-4.8 (42.9)	P vs. SA	0.014	-11.5 (-21.6 to -1.3)
Change at 12 months (SD)	8.0 (40.4)	-2.0 (46.3)	-6.0 (36.6)	W vs. P	0.207	5.2 (-2.9 to 13.4)
Change at 24 months (SD)	-1.3 (38.1)	-6.7 (41.6)	-6.2 (43.5)			

^a^Negative change values indicate a decrease from baseline.

^b^n’s reflect group sizes at baseline, excluding those having no followup data (n = 17).

^c^Pairwise comparisons of groups based on estimated marginal means adjusted for age and baseline value of outcome using linear mixed model methods to conduct patterned covariance analysis.

^d^Comparisons of Web-based and Print-mailed with Standard Advice were one-tailed, Web-based with Print-mailed was two-tailed, each tested at α = .05.

^e^Effect was not in the hypothesized direction.

**Table 3:** **Mean (SD) change from Baseline** ^**a**^ **for Eating and Activity Behavioral Markers by Group**

	**Observed Means**			**Estimated Mean Difference over 24 months** ^**c**^		
	**Standard advice (SA)**	**Web-based (W)**	**Print-mailed (P)**	**Pairwise**		
	**n=53** ^**b**^	**n=108** ^**b**^	**n=111** ^**b**^	**Comparison**	**p-value** ^**d**^	**Estimate (95% CI)**
	**Mean (SD)**	**Mean (SD)**	**Mean (SD)**			
Weekly minutes of moderate or greater physical activity						
Baseline	266.4 (196.1)	265.3 (269.4)	227.6 (238.6)	W vs. SA	NS	18.6 (-46.1 to 83.3)
Change at 6 months (SD)	64.6 (274.7)	81.7 (343.9)	135.8 (329.1)	P vs. SA	NS	12.7 (-52.3 to 77.8)
Change at 12 months (SD)	103.9 (386.4)	91.3 (349.9)	96.0 (262.3)	W vs. P	NS	5.9 (-46.2 to 58.0)
Change at 18 months (SD)	17.0 (258.4)	96.8 (352.7)	120.6 (349.4)			
Change at 24 months (SD)	44.2 (306.8)	73.3 (362.3)	79.5 (390.0)			
Kcal expended daily						
Baseline	2159.4 (374.4)	2059.5 (371.8)	2131.6 (435.6)	W vs. SA	NS	-38.0 (-95.3 to 19.4)
Change at 6 months (SD)	13.1 (182.8)	7.6 (198.7)	48.5 (242.5)	P vs. SA	NS	1.8 (-55.6 to 59.2)
Change at 12 months (SD)	65.8 (287.4)	21.7 (211.7)	43.8 (187.8)	W vs. P	NS	-39.8 (-85.9 to 6.4)
Change at 18 months (SD)	26.4 (203.5)	22.5 (226.8)	63.0 (276.5)			
Change at 24 months (SD)	34.7 (193.1)	0.5 (243.5)	60.3 (326.6)			
Kcal intake daily						
Baseline	1702.6 (627.3)	1644.9 (559.0)	1740.3 (613.6)	W vs. SA	0.024^e^	113.4 (1.2 to 225.7)
Change at 6 months (SD)	-207.4 (393.1)	-63.3 (443.7)	-94.6 (520.2)	P vs. SA	0.027^e^	110.9 (-1.5 to 223.4)
Change at 12 months (SD)	-227.2 (466.5)	-94.9 (505.7)	-143.1 (513.3)	W vs. P	NS	2.5 (-88.1 to 93.1)
Change at 18 months (SD)	-256.8 (426.0)	-120.1 (524.9)	-210.0 (514.5)			
Change at 24 months (SD)	-211.4 (421.3)	-169.5 (433.9)	-182.0 (594.9)			
Percent daily calories from fat						
Baseline	36.9 (5.6)	36.6 (6.2)	36.4 (4.6)	W vs. SA	0.018	-1.4 (-2.6 to -0.1)
Change at 6 months (SD)	-2.4 (5.7)	-3.8 (5.2)	-3.7 (5.1)	P v. SA	0.03	-1.2 (-2.5 to 0.1)
Change at 12 months (SD)	-3.1 (5.7)	-3.7 (5.0)	-3.5 (4.8)	W vs. P	NS	-0.1 (-1.2 to 0.9)
Change at 18 months (SD)	-1.5 (5.5)	-3.6 (5.1)	-3.3 (4.7)			
Change at 24 months (SD)	-1.8 (5.3)	-3.3 (5.4)	-2.8 (5.1)			
Percent daily calories from saturated fat						
Baseline	10.9 (1.9)	10.8 (2.3)	10.8 (1.9)	W vs. SA	0.049	-0.4 (-0.8 to 0.1)
Change at 6 months (SD)	-0.9 (1.9)	-1.4 (1.7)	-1.6 (2.0)	P vs. SA	0.013	-0.5 (-0.9 to -0.1)
Change at 12 months (SD)	-1.3 (2.0)	-1.3 (1.8)	-1.4 (1.9)	W vs. P	NS	0.1 (-0.2 to 0.5)
Change at 18 months (SD)	-0.6 (1.8)	-1.3 (1.8)	-1.2 (1.8)			
Change at 24 months (SD)	-0.7 (1.8)	-1.1 (1.9)	-1.2 (2.0)			
Fruit and vegetable daily servings						
Baseline	4.9 (2.1)	4.8 (2.6)	5.1 (2.3)	W vs. SA	0.008	0.8 (0.1 to 1.4)
Change at 6 months (SD)	0.8 (2.1)	1.8 (2.2)	2.1 (2.4)	P vs. SA	<0.005	1.2 (0.6 to 1.8)
Change at 12 months (SD)	0.9 (2.5)	1.7 (2.4)	2.0 (2.5)	W vs. P	NS	-0.4 (-0.9 to 0.1)
Change at 18 months (SD)	0.5 (2.1)	1.4 (2.6)	1.4 (2.7)			
Change at 24 months (SD)	0.9 (2.5)	1.0 (2.5)	1.6 (2.7)			
Low-fat dairy daily servings						
Baseline	1.3 (0.9)	1.4 (1.0)	1.5 (1.0)	W vs. SA	<0.001	0.3 (0.1 to 0.5)
Change at 6 months (SD)	-0.1 (0.6)	0.2 (0.9)	0.1 (0.7)	P vs. SA	0.002	0.3 (0.1 to 0.5)
Change at 12 months (SD)	-0.2 (0.6)	0.2 (1.0)	0.1 (0.7)	W vs. P	NS	0.1 (-0.1 to 0.2)
Change at 18 months (SD)	-0.2 (0.7)	0.0 (0.9)	0.0 (0.7)			
Change at 24 months (SD)	-0.1 (0.7)	0.0 (0.9)	0.1 (0.7)			
Sodium (mg/day)						
Baseline	2926 (1149)	2748 (1029)	3048 (1190)	W vs. SA	0.030^e^	206.2 (-8.7 to 421.1)
Change at 6 months (SD)	-368 (743)	-50 (849)	-251 (1007)	P vs. SA	0.026^e^	214.3 (-0.7 to 429.4)
Change at 12 months (SD)	-412 (870)	-140 (935)	-260 (988)	W vs. P	NS	-8.1 (-182.1 to 165.9)
Change at 18 months (SD)	-472 (794)	-208 (968)	-355 (1031)			
Change at 24 months (SD)	-332 (856)	-309 (818)	-373 (1091)			

^a^Negative change values indicate a decrease from baseline.

^b^n’s reflect group sizes at baseline, excluding those having no followup data (n = 17).

^c^Pairwise comparisons of groups based on estimated marginal means adjusted for age and baseline value of outcome using linear mixed model methods to conduct patterned covariance analysis.

^d^Comparisons of Web-based and Print-mailed with Standard Advice were one-tailed, Web-based with Print-mailed was two-tailed, each tested at α = .05.

^e^Effect was not in the hypothesized direction.

All tests of the Group X Time interaction were non-significant, indicating that the differences among groups did not vary across time for any of the outcomes. Descriptively, all groups showed a general pattern of improvement over time on most outcomes. The exception was low fat dairy servings, which declined in the standard advice group.

Linear mixed model results of pairwise comparisons of estimated marginal mean change across the 24-month study, adjusted for age and baseline level, are summarized below. Consistent with the hypothesis, both the web-based group and print-mailed group improved significantly more than did the standard advice group on several dietary measures, with greater reductions in percent of calories from fat (p = .018 and p = .030) and from saturated fat (p = .049 and p = .013) and greater increases in servings of fruit and vegetables (p = .008 and p < .005) and low fat dairy (p < .001 and p = .002, respectively). The print-mailed group improved significantly more than the standard advice group on triglycerides (p = .014). Waist circumference also decreased significantly more in the web-based and print-mailed groups than in the standard advice group (p = .017 and p = .016, respectively). In addition, the web-based group showed a significantly greater decrease in BMI (however, the p-value for this comparison increased from .047 to .056 if a single outlier was deleted), a larger increase in fitness defined as estimated VO_2_max (p = .037), and significantly greater improvement in systolic blood pressure (p = .048) compared to the standard advice group. In the supplemental analysis without women who were prescribed antihypertension medication, none of the pairwise comparisons for systolic or diastolic blood pressure were significant (the p-value for web-based compared to standard advice changed from .048 to .124). The pattern of means remained the same, but mean differences between groups were slightly smaller for systolic blood pressure than the differences found in the primary analysis.

Analysis of the degree of change in the intervention groups compared to the standard advice group on kilocalorie intake (p = .024 and p = .027) and sodium (p = .030 and p = .026) indicated the standard advice group had greater decline on each of these outcomes, contrary to expectations. There were no significant differences between the web-based and print-mailed groups in change on any of the outcomes.

### Proportion meeting health outcome criteria

Table 4 displays the proportion in each group meeting normotensive status and eating or activity behavioral marker criteria and includes results of the analysis using generalized estimating equations. After the 12-month intervention, the proportions of women achieving normotensive status were 51% in standard advice, 49% in web-based, and 43% in print-mailed groups, respectively. After 24 months (12 months post-intervention), the proportions of women in the standard advice, web-based, and print-mailed groups that met normotensive status were 29%, 46%, and 31%, respectively. Over the 24-month trial, 16 women were prescribed antihypertensive medication, representing seven women in the standard advice group, four women in the web-based group, and five women in the print-mailed group. Omitting these women from the calculations, normotensive status was achieved by 49%, 49% and 45% at 12 months and by 33%, 47%, and 29% of the women in each group at 24 months.

**Table 4:** **Group Comparison of Frequency (Proportion) meeting Health Outcomes for Blood Pressure, Healthy Eating and Activity Behavioral Targets**

**Criteria**	**Standard**	**Web-**	**Print-**	**Odds ratio (95% CI)** ^**a**^		
	**Advice (SA)**	**based (W)**	**mailed (P)**	**W vs SA**	**P vs SA**	**W vs P**
Normotensive status						
6 months	21/53 (.40)	55/108 (.51)	47/111 (.42)	1.5 (0.7 to 3.1)	1.2 (0.6 to 2.5)	1.3 (0.7 to 2.3)
12 months	26/51 (.51)	51/105 (.49)	46/108 (.43)	0.8 (0.4 to 1.6)	0.8 (0.4 to 1.7)	1.0 (0.5 to 1.8)
18 months	21/48 (.44)	56/102 (.55)	42/103 (.41)	1.7 (0.7 to 3.6)	1.0 (0.5 to 2.2)	1.7 (0.9 to 3.1)
24 months	14/48 (.29)	48/104 (.46)	32/104 (.31)	2.1 (1.0 to 4.7)	1.3 (0.6 to 2.9)	1.7 (0.9 to 3.1)
Estimated marginal proportion^b^	0.39	0.47	0.39	p = .11	p = .48	p = .09
≥ 150 minutes weekly of moderate or greater intensity activity						
6 months	44/53 (.83)	87/108 (.81)	86/111 (.78)	0.8 (0.4 to 2.0)	0.7 (0.3 to 1.7)	1.2 (0.5 to 2.8)
12 months	37/51 (.73)	79/106 (.75)	79/106 (.73)	1.1 (0.5 to 2.5)	1.1 (0.5 to 2.3)	0.9 (0.4 to 1.9)
18 months	38/48 (.79)	85/102 (.83)	81/102 (.79)	1.3 (0.5 to 3.1)	0.9 (0.4 to 2.2)	0.8 (0.3 to 1.9)
24 months	34/48 (.71)	71/104 (.68)	67/103 (.65)	0.9 (0.4 to 2.0)	0.8 (0.4 to 1.8)	1.1 (0.5 to 2.3)
Estimated marginal proportion^b^	0.77	0.78	0.75	p = .41	p = .34^c^	p = .44
<27% daily calories from fat						
6 months	4/53 (.08)	17/108 (.16)	14/111 (.13)	2.3 (0.7 to 7.9)	1.8 (0.5 to 6.5)	0.4 (0.1 to 1.5)
12 months	4/51 (.08)	12/106 (.11)	12/109 (.11)	1.4 (0.4 to 5.1)	1.4 (0.4 to 5.3)	0.7 (0.2 to 2.7)
18 months	5/48 (.10)	15/102 (.15)	13/103 (.13)	1.5 (0.5 to 4.9)	1.4 (0.4 to 4.6)	0.6 (0.2 to 2.1)
24 months	4/48 (.08)	13/104 (.13)	11/104 (.11)	1.6 (0.5 to 5.6)	1.4 (0.4 to 5.1)	0.6 (0.2 to 2.2)
Estimated marginal proportion^b^	0.05	0.09	0.08	p = .10	p = .15	p = .76
<7% daily calories from saturated fat						
6 months	4/53 (.08)	8/108 (.07)	5/111 (.05)	0.7 (0.2 to 2.9)	0.6 (0.1 to 2.6)	1.5 (0.4 to 6.4)
12 months	3/51 (.06)	7/106 (.07)	9/109 (.08)	0.9 (0.2 to 4.1)	1.3 (0.3 to 5.8)	1.2 (0.2 to 5.4)
18 months	3/48 (.06)	9/102 (.09)	8/103 (.08)	1.3 (0.3 to 5.7)	1.4 (0.3 to 6.0)	0.8 (0.2 to 3.2)
24 months	1/48 (.02)	9/104 (.09)	5/104 (.05)	4.5 (0.5 to 41.3)	2.9 (0.3 to 28.6)	0.2 (.02 to 2.0)
Estimated marginal proportion^b^	0.02	0.03	0.03	p = .27	p = .30	p = .89
>8 daily servings of fruit and vegetables						
6 months	8/53 (.15)	32/108 (.30)	38/111 (.34)	2.9 (1.1 to 7.6)	3.3 (1.3 to 8.5)	0.3 (0.1 to 0.9)
12 months	7/51 (.14)	32/106 (.30)	38/109 (.35)	3.3 (1.2 to 8.9)	4.2 (1.6 to 11.2)	0.3 (0.1 to 0.8)
18 months	5/48 (.10)	30/102 (.29)	27/103 (.26)	4.5 (1.5 to 13.8)	3.9 (1.3 to 11.9)	0.2 (0.1 to 0.7)
24 months	7/48 (.15)	25/104 (.24)	32/104 (.31)	2.0 (0.8 to 5.4)	2.9 (1.1 to 7.6)	0.5 (0.2 to 1.3)
Estimated marginal proportion^b^	0.10	0.25	0.27	p < .005	p < .005	p = .63
> 2 daily servings of low-fat dairy						
6 months	10/53 (.19)	37/108 (.34)	29/111 (.26)	2.4 (0.9 to 6.4)	1.3 (0.5 to 3.6)	0.4 (0.2 to 1.1)
12 months	7/51 (.14)	38/106 (.36)	33/109 (.30)	4.7 (1.6 to 13.7)	2.9 (1.0 to 8.6)	0.2 (0.1 to 0.6)
18 months	6/48 (.13)	25/102 (.25)	23/103 (.22)	2.6 (0.8 to 8.1)	2.2 (0.7 to 7.2)	0.4 (0.1 to 1.2)
24 months	9/48 (.19)	24/104 (.23)	27/104 (.26)	1.1 (0.4 to 3.4)	1.5 (0.5 to 4.4)	0.9 (0.3 to 2.6)
Estimated marginal proportion^b^	0.11	0.24	0.20	p = .01	p = .05	p = .38
< 2400 mg of daily sodium						
6 months	22/53 (.42)	46/108 (.43)	48/111 (.43)	0.8 (0.4 to 1.8)	1.2 (0.5 to 2.6)	1.2 (0.5 to 2.6)
12 months	26/51 (.51)	49/106 (.46)	42/109 (.39)	0.7 (0.3 to 1.4)	0.5 (0.2 to 1.2)	1.5 (0.7 to 3.4)
18 months	29/48 (.60)	55/102 (.54)	47/103 (.46)	0.6 (0.3 to 1.5)	0.5 (0.2 to 1.3)	1.6 (0.7 to 3.7)
24 months	23/48 (.48)	57/104 (.55)	48/104 (.46)	1.4 (0.6 to 3.0)	1.1 (0.5 to 2.5)	0.7 (0.3 to 1.6)
Estimated marginal proportion^b^	0.48	0.43	0.43	p = .24^c^	p = .23^c^	p = .90

Note: GEE comparisons of Web-based and Print-mailed with Standard Advice were one-tailed, Web-based with Print-mailed was two-tailed, each tested at α = .05.

^a^Odds ratios calculated separately for each time point and adjusted for age and baseline outcome.

^b^Estimated marginal proportions from generalized estimating equations adjusted for age and baseline level of outcome.

^c^Effect was not in hypothesized direction.

The results of the analysis using generalized estimating equations showed a higher estimated proportion of women in the web-based group achieved normotensive status than in the print-mailed or standard advice groups, but differences were not significant (p = .109 and p = .088, respectively). Similar results were found in the supplemental analysis (p = .092 and p = .115, respectively). In addition, compared to the standard advice group, both web-based and print-mailed groups had significantly greater proportions of women meeting recommendations of a minimum of 8 daily servings of fruit and vegetables (both p < .005) and 2 servings of low fat dairy (p = .01 and p = .05). All Group X Time interactions were non-significant except for low fat dairy, for which the magnitude of differences among groups differed significantly over time (Wald χ^2^ (6) = 15.34, p = .018); however, the proportion in the standard advice group was consistently lower than in the other groups. In general, the proportions meeting criteria were quite low regardless of intervention. Exceptions were sodium consumption and normotensive status, each approaching half of the sample, and engaging in at least 150 minutes weekly of activity at moderate or greater intensity, which was reported by approximately 75% of all groups.

## Discussion

The *Wellness for Women: DASHing towards Health* clinical trial found many rural women with prehypertension were able to reduce blood pressure sufficiently to achieve normotensive status across group and time points. Although a higher estimated proportion of women in the web-based group (47%) achieved normotensive status than in the print-mailed (39%) or standard advice groups (39%), these proportions did not significantly differ. Conclusions were the same regardless of whether women who began taking antihypertension medications were included in the analysis. The relatively high proportions of women in each group who achieved normotensive status is of clinical importance, because individuals with prehypertension are associated with a 27% increase in all-cause and a 66% increase in cardiovascular disease mortality when compared to individuals who are normotensive [52].

The results of this community-based trial are encouraging and are consistent with the findings among participants in the PREMIER trial who had above-optimal blood pressure (defined as not being on antihypertensive medication and having a systolic blood pressure of 120–159 mm Hg and/or a diastolic blood pressure of 80 to 95 mm Hg), where normotensive status was achieved by 40% of individuals in the established behavioral intervention group and by 48% of individuals in the established behavioral intervention group plus the DASH [16]. Consistent with our hypotheses, both web-based and print-mailed tailored intervention groups accomplished greater improvements in measures of waist circumference and dietary measures compared to the standard advice group. These findings are consistent with a recent systematic review reporting positive effects for computer-tailored diet and activity interventions compared to generic interventions [22]. Specifically, rural women in both web-based and print-mailed groups had greater reductions in percent calories from fat and saturated fat and greater increases in servings of fruit and vegetables and low fat dairy than women receiving standard advice, and women in the web-based group demonstrated greater reductions in systolic blood pressure and BMI over time than women in the standard advice group.

Available studies that compared web-based and print-mailed methods of delivering tailored interventions used different populations, targeted one or more behaviors (diet or activity or both), and implemented different length and dose of interventions, making it difficult to make comparisons of their work with this study [22]. Several groups of researchers have compared the use and effects of both modes of delivery, suggesting that age, gender, socioeconomic factors, and health status may influence either the preference of use and/or outcomes of the intervention [22,53,54].

Our 12-month post-intervention outcomes showed the reductions in both systolic blood pressure and diastolic blood pressure of rural women to be within the range reported post-intervention in the DASH [55] and the PREMIER trials [16]. At 24 months (12 months post-intervention), the rural women in the standard advice and print-mailed groups demonstrated lower proportions of normotensive status than immediately post-time intervention, which is similar to findings in above-optimal participants in the PREMIER trial, where declines in blood pressure reduction occurred at 18 months (12 months post-intervention) [17].

Similar to the PREMIER studies [16,17], a high proportion of women in the standard advice group demonstrated a reduction of blood pressure to normotensive status, representing 40%, 51%, 44%, and 29% of women from baseline to 6, 12, 18, and 24 months, respectively (40%, 49%, 43%, and 33% if calculated without those taking antihypertension medication). This might be attributed to findings that the standard advice group showed greater reductions in sodium than the two intervention groups, with the estimated marginal proportions of women meeting the target of <2400 mg of daily sodium as 48% for standard advice group and 43% for both intervention groups. Studies have shown that low-salt intake can reduce blood pressure [56]. The reassessments, which were relatively frequent in order to obtain current data to guide message tailoring, may have had an unintended effect as intervention boosters. Further, this effect may have been stronger in the standard advice group, which lacked the feedback that logging provided to the other intervention groups. The literature suggests that interventions that incorporate frequent scheduled follow-up are generally more effective [57]. The PREMIER group also questioned whether frequent contacts made a difference in their control group and noted that highly motivated volunteers who are recruited in randomized trials that include standard advice groups may not be representative of the general population [16,17].

JNC-7 recommended weight loss for effective blood pressure reduction even without attaining normal BMI, and there was no change in this recommendation in the recently published JNC-8 [57]. A meta-analysis of 25 randomized clinical trials showed that a weight loss of one kg was associated with one mm Hg reduction in systolic blood pressure and diastolic blood pressure in individuals with prehypertension [58]. However, our interventions emphasized achieving dietary and activity behavioral targets and did not specifically address weight loss; results showed the weight of rural women remained stable (±2 pounds) in all groups over time.

The web-based group had the highest estimated proportion of women attaining normotensive status and also showed a greater reduction in waist circumference and a larger increase in estimated cardiorespiratory fitness (VO_2_max) compared to the standard advice group, suggesting that women in this group may have been more likely to participate in the physical activity component of the intervention. Numerous studies demonstrate the influence of cardiorespiratory fitness on reducing blood pressure, independent of weight loss [59]. Increased activity, resulting in increased fitness and reduced abdominal fatness, was shown to make an additive contribution toward blood pressure reduction [60]. The accuracy of the self-reported physical activity may be suspect, however, especially given that all group means on that outcome exceeded 250 minutes per week at baseline, far above what is generally reported for this population. This might be attributed to the challenges that sedentary individuals face in accurate recall of their participation in moderate or greater physical activity [61]. Objective measurement of physical activity using accelerometers would have enhanced the analysis about the women’s level of activity.

The blood lipid levels remained stable across all groups over 24 months, with observed lipid values in the normal or desirable ranges across all groups (HDL >50 mg/dL, LDL <129 mg/dL, triglycerides <150 mg/dL, and total cholesterol <200 mg/dL) with one exception of web-based group having mean (SD) of 201.9 (34.8) mg/dL. Although differences were observed in triglycerides between the print-mailed group and control group over 24 months, this difference was not clinically meaningful as the triglyceride levels remained in the normal range. The lack of change may have been influenced by the women’s overall healthy lipid profile at baseline, thus with little room for improvement.

Women who volunteered for the study had more education and were of higher socio-economic status than the general population, which may limit the generalizability of the results to the broader population. The rural women, because of their familiarity with other women in their areas of residence, may have shared information with women assigned to other groups, and contamination between groups may have occurred. This community-based intervention did not track the degree to which women participated in each of the components of the intervention nor the compliance of women’s participation in their assigned groups, which are limitations. Subsequent to the completion of the data collection for this trial, an extension of The Consolidated Standard of Reporting Trials [CONSORT] was developed to provide guidance for researchers when developing and reporting E-health interventions, especially as related to tracking web use (eg. logins, logfile analysis), which would have further informed the outcomes of this study [62].

The strengths of this study included the successful recruitment and retention of a large cohort of rural women to participate in a 24-month community-based trial that compared standard advice with tailored web-based and print-mailed lifestyle interventions for both eating and activity behavior change, with findings that nearly half of each group met normotensive status. As reimbursement is limited for preventive services such as lifestyle therapies for diet, physical activity and blood pressure monitoring, future studies might focus on cost-effectiveness of implementing this type of community-based approach for prehypertension management.

## Conclusions

Finding effective methods to reach and influence behavior change in rural women with prehypertension, who are at high risk for developing hypertension, is a public health challenge. This study supports that rural women with prehypertension are willing to participate in behavior-change interventions via distance-delivery methods with the goal of reducing their blood pressure. Women in both intervention groups demonstrated greater improvements in reducing waist circumference and improving dietary measures than the standard advice group; women in the web-based group also achieved greater improvements in reducing systolic blood pressure and estimated cardiovascular fitness than the standard advice group. Rural women were able to make and sustain behavior changes with favorable results of nearly one-half of women in each group attaining normotensive blood pressure.

## Abbreviations

JNC 7 refers: Joint national committee on prevention, detection, evaluation and treatment of high blood pressure – seventh report
DASH: Dietary approaches to stop hypertension
HPM: Health promotion model
JNC 8: Joint national committee on prevention, detection, evaluation and treatment of high blood pressure – eighth report
